# 2034 Effect of balanced crystalloids on renal outcomes among critically ill adults does not differ from 0.9% saline across baseline risk of renal outcomes

**DOI:** 10.1017/cts.2018.165

**Published:** 2018-11-21

**Authors:** Andrew C. McKown, Todd W. Rice, Matthew W. Semler

**Affiliations:** Vanderbilt University Medical Center

## Abstract

OBJECTIVES/SPECIFIC AIMS: Traditional clinical trials typically enroll a homogenous population to test the efficacy of an intervention. Pragmatic trials deliberately enroll a more diverse population to enhance generalizability, but doing so may increase heterogeneity of treatment effect among subpopulations. For example, the effect of a treatment on an outcome may vary based on patients’ sex, comorbidities, or baseline risk of experiencing the outcome. We hypothesized that heterogeneity of treatment effect by baseline risk for the outcome could be demonstrated in a large pragmatic clinical trial. METHODS/STUDY POPULATION: We performed a prespecified secondary analysis of a recent pragmatic trial comparing balanced crystalloids Versus 0.9% saline among critically ill adults. The primary endpoint of the trial was major adverse kidney events within 30 days of ICU admission, censored at hospital discharge (MAKE30). MAKE30 is a composite outcome of all-cause mortality, new renal replacement therapy, or persistent renal dysfunction. Using a previously published model with high predictive accuracy for MAKE30 (area under the curve=0.903), we calculated the baseline risk of MAKE30 for all trial participants. We then developed a logistic regression model for MAKE30 with independent covariates of fluid group assignment, baseline risk of MAKE30 as a nonlinear continuous variable, and the interaction between group assignment and MAKE30 baseline risk. RESULTS/ANTICIPATED RESULTS: Among 15,802 patients from 5 intensive care units enrolled in the original trial, 126 had missing variables for predicted risk of MAKE30. Mean predicted risk of MAKE30 among all patients was 15.4%; median was 4.4% (interquartile range 2.2%–17.1%). Predicted risk of MAKE30 did not significantly differ between groups (*p*=0.61 by Mann-Whitney *U*-test). The incidence of MAKE30 in the trial was 14.9%, and the prediction model was well-calibrated overall (AUC=0.891). In a logistic regression model examining the interaction between group assignment and predicted risk of MAKE30, group assignment significantly affected MAKE30 (odds ratio saline:balanced 1.13, 95% CI: 1.02–1.27, *p*=0.02), but we observed no interaction between the effect of group assignment on MAKE30 and patients’ predicted risk of MAKE30 at baseline (*p*=0.66 for interaction term). DISCUSSION/SIGNIFICANCE OF IMPACT: In a large pragmatic trial demonstrating a significant difference in the primary outcome of MAKE30 between balanced crystalloids and saline, a previously published model accurately predicted MAKE30 using baseline factors. However, contrary to our hypothesis, the baseline risk of MAKE30 did not modify the effect of fluid group on the observed incidence of MAKE30. Our analysis could not account for unmeasured confounders and may be underpowered to detect a significant interaction. Our findings suggest that the impact of balanced crystalloids versus normal saline on renal outcomes in critically patients is consistent across all levels of risk.

